# Mamma Succenturiata

**Published:** 1873-11

**Authors:** Matthews Duncan


Mamma Succenturiata—*Dr. Matthews Duncan.*—Gland in the axilla, about the size of a chestnut, just beyond the margin of the tendon of pectoralis major. It is deeply situated, being prominent and visible only when congested. When enlarged it causes great incovenience, but pressure on the gland produces a stream of milk, which flows from it by a minute opening situated at its center, and thus relief is procured. When the right mamma is enlarged through lacteal engorgement, this axillary gland is likewise increased in size; and when the right mamma is flaccid, so also is this axillary gland. No anatomical connection between the mamma and the axillary gland can be discovered. The secretion from the axillary gland, on being examined microscopically, is found to present the character of milk, consisting of fat globules of various sizes, colostrum, corpuscles, etc. It is similar to that which is obtained from the mammæ of the patient in every respect.

